# Clinical Characteristics of the Patient With Unmeasurable Ankle-Brachial Index in Endovascular Treatment

**DOI:** 10.7759/cureus.39705

**Published:** 2023-05-30

**Authors:** Yoh Arita, Kosuke Hirose, Yuto Suetani, Kana Shichijo, Shohei Yamamoto, Tomoki Fukui, Nobuyuki Ogasawara

**Affiliations:** 1 Department of Cardiology, Japan Community Healthcare Organization Osaka Hospital, Osaka, JPN

**Keywords:** tibial vessel runoff, poor collateral vessels, lower extremity arterial disease, endovascular treatment, calcification, ankle-brachial index

## Abstract

Introduction

Ankle-brachial index (ABI) is an important indicator to diagnose lower extremity arterial disease (LEAD). However, patients with unmeasurable ABI are sometimes excluded from the analysis and their clinical characteristics are poorly understood.

Methods

One hundred twenty-two consecutive Japanese subjects (mean age, 72 years), who underwent successful endovascular treatment (EVT) for lower extremity arteries at our hospital were retrospectively studied.

Results

Of the 122 patients, 23 (19%) patients presented an unmeasurable ABI before EVT. Five of 23 (22%) had still an unmeasurable ABI one day after EVT. Comorbidities including hypertension, diabetes, dyslipidemia, hemodialysis, smoking, ischemic heart disease, atrial fibrillation, and past-EVT history were not different between ABI measurable and unmeasurable patients. However, patients with unmeasurable ABI presented a significantly higher degree of Rutherford category and a smaller number of tibial vessel runoff than patients with measurable ABI before EVT (p<0.05 and p<0.01, respectively). There was no difference in the lesion site between the two groups. The event rate including all-cause mortality, re-EVT, lower limb amputation, and bypass surgery did not differ between two groups four years after EVT. ABI after four years of initial EVT did not differ between pre-EVT measurable and unmeasurable patients (0.96 vs. 0.84, p=0.48).

Conclusions

Patients with unmeasurable ABI before EVT were characterized by higher degree of Rutherford categorization and a small number of tibial vessel runoff, but there was no significant difference in outcomes during the follow-up period.

## Introduction

Ankle-brachial index (ABI) is a pivotal indicator to diagnose lower extremity arterial disease (LEAD). Lower extremity blood pressure in patients with unmeasurable ABI has been reported to be less than 34 mmHg using Doppler ultrasound [1]. Patients with unmeasurable ABI are sometimes excluded from the analysis [2]. Alternatively, there are many cases where there is no description of how ABI unmeasurable cases were statistically processed. Therefore, the clinical characteristics of patients with unmeasurable ABI are poorly understood. In this study, we investigated in detail the patients' background and clinical outcomes presenting unmeasurable ABI for endovascular treatment (EVT).

## Materials and methods

One hundred twenty-two consecutive Japanese subjects (mean age, 72 years), who underwent successful EVT for lower extremity arteries at our hospital from January 2017 to December 2018, were retrospectively studied. The ABI data were obtained using an automated oscillometric device (VP-1000; Omron Healthcare Co., Kyoto, Japan) [3]. The lesion length was classified as follows: focal, ≤1 cm; short, >1 and <5 cm; intermediate, ≥5 and <15 cm; and long, ≥15 cm [4]. The extent of lesion calcification was classified according to the peripheral arterial calcium scoring system (PACSS; Grade 0, no visible calcification; Grade 1, unilateral calcification, <5 cm; Grade 2, unilateral calcification, ≥5 cm; Grade 3, bilateral calcification, <5 cm; and Grade 4, bilateral calcification, ≥5 cm) [5]. The collateral score was defined by Jenali Collateral Scoring System [6]. Statistical analyses were performed using EZR software [7]. This study complied with the ethical standards of the responsible institution on human subjects as well as with the Helsinki Declaration.

## Results

**Figure 1:**
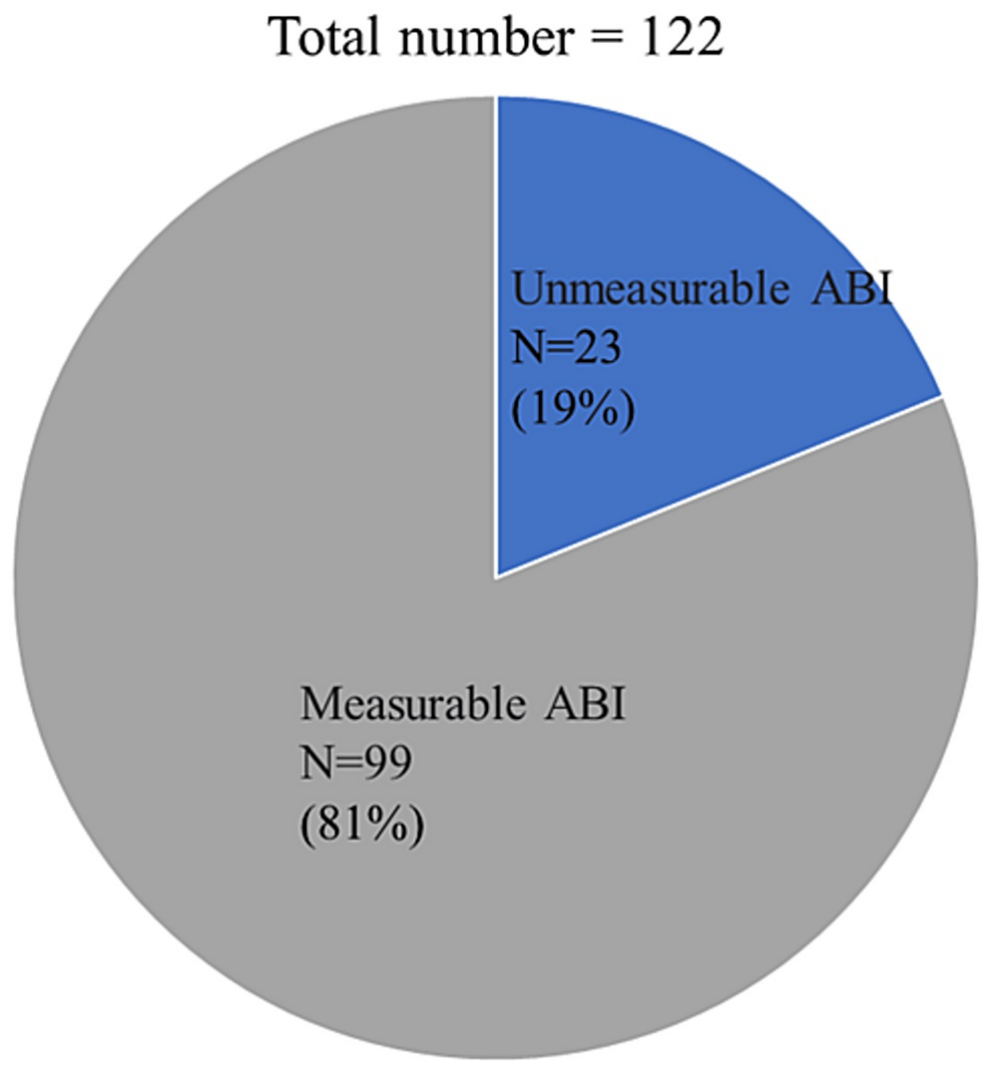
Percentage of patients with unmeasurable ABI before EVT. Of the 122 patients, 23 (19%) presented unmeasurable ABI.

Of the 122 patients, 23 (19%) patients presented an unmeasurable ABI before EVT (Figure 1).

The mean ABI of ABI measurable patients was 0.71 ± 0.18. Five of 23 (22%) had still an unmeasurable ABI one day after EVT. Patient characteristics are summarized in Table 1.

**Table 1:** Patient characteristics Data are expressed as the mean ± SD or number (%) ABI, ankle-brachial index; AF, atrial fibrillation; BMI, body mass index; CLI, critical limb ischemia; DM, diabetes mellitus; DL, dyslipidemia; EVT, endovascular treatment; HD, hemodialysis; HT, hypertension; IHD, ischemic heart disease; N/A, not available.

	Total	Measurable ABI	Unmeasurable ABI	P-value
Number	122	99	23	
ABI	N/A	0.71 ± 0.18	N/A	
Demographic				
Age, years	72.8 ± 7.9	73.3 ± 7.4	70.7 ± 9.7	0.15
Male	89 (73%)	73 (74%)	16 (70%)	0.79
Height, m	1.59 ± 0.09	1.59 ± 0.08	1.62 ± 0.12	0.17
Weight, kg	58.7 ± 13.7	58.6 ± 13.1	59.4 ± 16.3	0.80
BMI, kg/m2	22.8 ± 4.3	23.0 ± 4.4	22.3 ± 4.3	0.54
Medical history				
HT	96 (79%)	76 (77%)	20 (87%)	0.4
DM	70 (57%)	57 (58%)	13 (57%)	1
DL	77 (63%)	64 (64%)	13 (57%)	0.48
IHD	69 (57%)	57 (58%)	12 (52%)	0.64
HD	48 (39%)	36 (36%)	12 (52%)	0.23
AF	23 (19%)	16 (16%)	7 (30%)	0.14
past-EVT	70 (57%)	57 (58%)	13 (57%)	1
Current/Past Smoking	80 (66%)/6 (5%)	64 (65%)/6 (6%)	16(70%)/0 (0%)	0.71
Clinical presentation				
Fontaine stage				< 0.01
2	57 (47%)	54 (55%)	3 (13%)	
3	10 (8%)	5 (5%)	5 (22%)	
4	55 (45%)	40 (40%)	15 (65%)	
Rutherford category				< 0.01
2	42 (34%)	40 (40%)	2 (9%)	
3	16 (13%)	14 (14%)	2 (9%)	
4	11 (9%)	6 (6%)	5 (22%)	
5	48 (39%)	35 (35%)	13 (57%)	
6	5 (4%)	4 (4%)	1 (4%)	
CLI	65 (53%)	46 (47%)	19 (83%)	< 0.01

In brief, comorbidities including hypertension, diabetes, dyslipidemia, ischemic heart disease, hemodialysis, atrial fibrillation, past-EVT history and smoking were not different between ABI measurable and unmeasurable patients. However, patients with unmeasurable ABI presented significantly higher degree of Fontaine stage and Rutherford category eventually presenting critical limb ischemia (CLI). Lesion characteristics are summarized in Table 2.

**Table 2:** Lesion characteristics BTK, below the knee; CTO, chronic total occlusion; DCB, drug-coated balloon; PACSS, peripheral arterial calcium scoring system; POBA, plain old balloon angioplasty; POP, popliteal artery; SFA, superficial femoral artery.

	Total	Measurable ABI	Unmeasurable ABI	P-value
	(n=122)	(n=99)	(n=23)	
Lesions				0.2
Iliac	21 (17%)	20 (20%)	1 (4%)	
SFA-POP	77 (63%)	59 (60%)	18 (78%)	
BTK	20 (16%)	16 (16%)	4 (17%)	
Bypass graft	4 (3%)	4 (4%)	0 (0%)	
Lesion length				0.37
Focal, ≤1 cm	15 (12%)	13(13%)	2 (9%)	
Short, >1 and <5 cm	44 (36%)	38 (38%)	6 (26%)	
Intermediate, ≥5 and <15 cm	40 (33%)	32 (32%)	8 (35%)	
Long, ≥15 cm	23 (19%)	16 (16%)	7 (30%)	
PACSS grade				0.76
0	12 (10%)	11 (11%)	1 (4%)	
1	37 (30%)	30 (30%)	7 (30%)	
2	26 (21%)	20 (20%)	6 (26%)	
3	32 (26%)	27 (27%)	5 (22%)	
4	15 (12%)	11 (11%)	4 (17%)	
CTO	44 (36%)	32 (32%)	12 (52%)	0.09
Tibial vessel runoff				<0.01
0	14 (11%)	4 (4%)	10 (43%)	
1	29 (24%)	20 (20%)	9 (39%)	
2	40 (33%)	37 (37%)	3 (13%)	
3	39 (32%)	38 (38%)	1 (4%)	
Collateral score				<0.01
0	58 (48%)	55 (56%)	3 (13%)	
1	19 (16%)	16 (16%)	3 (13%)	
2	23 (19%)	13 (13%)	10 (43%)	
3	22 (18%)	15 (15%)	7 (30%)	
Procedure				0.61
POBA	69 (57%)	58 (59%)	11 (48%)	
DCB	9 (7%)	7 (7%)	2 (9%)	
Stent	44 (36%)	34 (34%)	10 (43%)	

Lesion site (Iliac, superficial femoral artery to popliteal artery, below the knee and bypass graft), lesion length, chronic total occlusion, and PACSS grade were not different between two groups. The patients with unmeasurable ABI had significantly smaller number of tibial vessel runoff than patients with measurable patients before EVT (p<0.01). Moreover, the patients with unmeasurable ABI had significantly higher degree of collateral score indicating poor collateral vessels. Procedures including plain old balloon angioplasty, drug-coated balloon angioplasty and stent implantation were not different between two groups. Independent predictors of unmeasurable ABI were poor tibial vessel runoff (vessel 0-1) (odds ratio (OR), 6.98; 95% CI, 1.60-30.4; P<0.01) and poor collateral (Score 2-3) (OR, 4.66, 95% CI, 1.21-17.9; P=0.02) (Table 3).

**Table 3:** Logistic regression analysis for the unmeasurable ABI CLI, critical limb ischemia; CTO, chronic total occlusion; HD, hemodialysis

Variables	Multivariate	
	Odds ratio [95%CI]	P-value
HD	1.07 [0.27-4.15]	0.92
CLI	4.86 [0.90-26.1]	0.06
CTO	2.10 [0.58-7.53]	0.25
Long lesion ≥15cm	2.18 [0.56-8.47]	0.26
Poor tibial vessel runoff (vessel 0-1)	6.98 [1.60-30.4]	<0.01
Poor collateral (Score 2-3)	4.66 [1.21-17.9]	0.02
Severe calcification (PACSS 3-4)	0.20 [0.04-0.84]	0.02

Conversely, severe calcification (PACSS 3-4) was found to negatively predict unmeasurable ABI (OR, 0.20, 95% CI, 0.04-0.84; P=0.02). EVT procedure such as plain old balloon angioplasty, drug-coated balloon angioplasty and stent implantation was not different between two groups.

Clinical outcomes were summarized in Table 4. The event rate including death, re-EVT, lower limb amputation, and bypass surgery did not differ between two groups four years after EVT. ABI after four years from initial EVT did not differ between pre-EVT measurable and unmeasurable patients (0.96 vs. 0.84, p=0.48).

**Table 4:** Clinical outcomes at four years Data are expressed as the mean ± SD or number (%). ABI, ankle-brachial index; EVT, endovascular treatment.

	Total	Measurable ABI	Unmeasurable ABI	P-value
	(n=122)	(n=99)	(n=23)	
Death	31 (25%)	25 (25%)	6 (27%)	1
Amputation	9 (7%)	6 (6%)	3 (13%)	0.2
Bypass	2 (2%)	1 (1%)	1 (4%)	0.34
Re-EVT	45 (37%)	37 (37%)	8 (35%)	1
ABI	0.95 ± 0.62	0.94 ± 0.66	0.84 ± 0.27	0.48

## Discussion

In patients with a history or physical examination findings suggestive of LEAD, the resting ABI, with or without segmental pressures and waveforms, is recommended to establish the diagnosis [8]. Resting ABI results should be reported as abnormal (ABI ≤0.90), borderline (ABI 0.91-0.99), normal (1.00-1.40), or noncompressible (ABI >1.40). Values >1.40 indicate that the arteries were not able to be compressed, which is more common among individuals with diabetes mellitus and/or advanced chronic kidney disease. Since ABI measurement by the oscillometric method is affected by vascular calcification, the optimal cut-off value for dialysis patients is less than 1.05 or less than 1.06 unlike the general population [9,10]. In this study, there were no patients with ABI >1.40 before and after EVT.

In the previous study, 6% of LEAD patients with intermittent claudication had an unmeasurable ABI [1]. Our results showed that 19% of LEAD patients demonstrated unmeasurable ABI, which is higher than previously reported. Our patients also included patients with CLI, which may be attributed to the more severe LEAD patient background.

Patients with unmeasurable ABI prior to EVT were characterized by a higher degree of Fontaine stage and Rutherford categorization. In the previous study, chronic limb-threatening ischemia patients with higher ischemia grading in wound, ischemia, and foot infection classification presented lower ABI [11]. Our results were consistent with previous reports.

The patients with unmeasurable ABI had a significantly smaller number of tibial vessel runoff and a higher degree of collateral score. These findings were consistent since ABI indicates the presence of stenotic or obstructive lesions in major arteries proximal to the ankle and the degree of collateral circulation compensation [12].

In this study, multivariate analysis was performed to examine the determinants of unmeasurable ABI. Independent predictors of unmeasurable ABI were poor tibial vessel runoff and poor collateral vessels. However, severe calcification (PACSS 3-4) was found to negatively predict unmeasurable ABI. These results were consistent with the previous study since calcifications of the superficial femoral artery and below-knee arteries were negatively correlated with ABI [10].

The event rate including death, re-EVT, lower limb amputation, and bypass surgery did not differ between the two groups four years after EVT. This fact indicated that even if ABI was not measurable before EVT, the prognosis was favorable if EVT was successful. On the other hand, this result may be due to the limitations of this study, such as selection bias due to its retrospective nature, the small number of patients in a single hospital, and the limited and incomplete follow-up period. Further studies are required to validate our results.

## Conclusions

Patients with unmeasurable ABI prior to EVT were characterized by higher degree of Fontaine stage and Rutherford categorization, small number of tibial vessel runoff and poor collateral vessels. Severe calcification negatively predicted unmeasurable ABI. There was no significant difference in outcomes during the follow-up period.
